# Comparison Between Core Set Selection Methods Using Different Illumina Marker Platforms: A Case Study of Assessment of Diversity in Wheat

**DOI:** 10.3389/fpls.2020.01040

**Published:** 2020-07-09

**Authors:** Behnaz Soleimani, Heike Lehnert, Jens Keilwagen, Joerg Plieske, Frank Ordon, Sara Naseri Rad, Martin Ganal, Sebastian Beier, Dragan Perovic

**Affiliations:** ^1^ Federal Research Centre for Cultivated Plants, Institute for Resistance Research and Stress Tolerance, Julius Kuehn Institute, Quedlinburg, Germany; ^2^ Institute for Biosafety in Plant Biotechnology, Julius Kuehn Institute, Quedlinburg, Germany; ^3^ TraitGenetics GmbH, Gatersleben, Germany; ^4^ Department of Physiology and Cell Biology, Leibniz Institute of Plant Genetics and Crop Plant Research (IPK), Seeland, Germany; ^5^ Department of Breeding Research, Leibniz Institute of Plant Genetics and Crop Plant Research (IPK), Seeland, Germany

**Keywords:** molecular marker, *Triticum aestivum*, k-medoids, core set, 90K–15K-iSelect Illumina arrays, SNP

## Abstract

Collections of plant genetic resources stored in genebanks are an important source of genetic diversity for improvement in plant breeding programs and for conservation of natural variation. The establishment of reduced representative collections from a large set of genotypes is a valuable tool that provides cost-effective access to the diversity present in the whole set. Software like Core Hunter 3 is available to generate high quality core sets. In addition, general clustering approaches, *e.g.*, *k*-medoids, are available to subdivide a large data set into small groups with maximum genetic diversity between groups.

Illumina genotyping platforms are a very efficient tool for the assessment of genetic diversity of plant genetic resources. The accumulation of genotyping data over time using commercial genotyping platforms raises the question of how such huge amount of information can be efficiently used for creating core collections. In the present study, after developing a 15K wheat Infinium array with 12,908 SNPs and genotyping a set of 479 hexaploid winter wheat lines (*Triticum aestivum*), a larger data set was created by merging 411 lines previously genotyped with the 90K iSelect array. Overlaying the markers from the 15K and 90K arrays enabled the identification of a common set of 12,806 markers, suggesting that the 15K array is a valuable and cost-effective resource for plant breeding programs.

Finally, we selected genetically diverse core sets out of these 890 wheat genotypes derived from five collections based on the common markers from the 15K and 90K SNP arrays. Two different approaches, *k*-medoids and Core Hunter 3 were compared,and *k*-medoids was identified as an efficient method for selecting small core sets out of a large collection of genotypes while retaining the genetic diversity of the original population.

## Introduction

Germplasm collections are an important source of natural genetic diversity and provide a source of novel traits for sustainable crop improvement (Wang et al., 2018). However, genebanks need to balance between storing and regenerating large collections with limited resources with respect to storage capacity and monetary constraints. Frankel (1984) introduced the term core collection as a concept. A core collection is a subset of accessions which were selected by eliminating closely related samples while still capturing the genetic diversity of the original set of accessions. Therefore, a core collection ideally represents the genetic diversity of the entire collection. Providing core collections with maximum genetic variation facilitates efficient management and utilization of genetic diversity (Brown, 1989; van Heerwaarden et al., 2013) and is an efficient method for characterizing and using genetic resources of crop plants without the need to sample the entire collection (Jeong et al., 2017). Originally, phenotypic data containing both morphological and agronomical traits were used to create core collections, whereas nowadays molecular markers as neutral tools for measuring genetic variation have become the tool of choice.

There are currently three different strategies for generating a core collection from a large population using molecular marker data (Odong et al., 2013). Firstly, it is possible to build up a core collection that represents the individual accessions (CC-I), *e.g.*, a uniform representation of the original population. Second, it is possible to select a core collection based on accessions that represents the distribution of all relevant traits (CC-D), *e.g.*, if the majority of the original population contains allele A at a given locus, then the core collection should imitate this behavior. Thirdly, accessions can be selected that represent the extremes of all relevant traits (CC-X), *e.g.*, different entries into the core collection should be as diverse as possible with regard to the selected traits. Depending on which strategy is used, there are disadvantages in terms of working with the whole population. For example, trait customized core collections (CC-X), which aim to maximize diversity for that particular trait, would be better suited to finding rare alleles than a core collection that is designed to represent the original population (CC-I). The loss of rare alleles, especially in plant and resistance breeding, is one of the main concerns when working with core collections (Odong et al., 2013).

The quality of core set selection can be evaluated by using a variety of mathematical measures. De Beukelaer et al. (2018) explained that distance-based measures are attractive because they are easy to understand and take into account both the diversity within the core set and representativeness of accessions from the entire collection. Nevertheless, pairwise distances are required to be aggregated in suitable ways to evaluate the quality of a selected core set. One such aggregation, which is often used, is to calculate the average of pairwise distances to obtain an estimate of the quality of the core set (De Beukelaer et al., 2018). The interpretation of the result depends strongly on the defined purpose of the core collection. While it might be advantageous for core collections built up with the aim of conserving extremely rare alleles (CC-X) and therefore aiming at a maximum of the average pairwise genetic distance, a core collection built up for a uniform representation of the population (CC-I) would want to minimize the average pairwise genetic distance. Odong et al. (2013) proposed different criteria for the evaluation of core collections. They defined a way to estimate the quality of the core set selection process and introduced two new distance-based metrics. These two metrics were also used in the study by De Beukelaer et al. (2018) to evaluate the quality of core collections in rice, coconut, maize and pea for various tools. Core Hunter 3 was able to convince particularly through its flexibility to combine different methods. The evaluation metrics used showed that Core Hunter 3 core collections were always competitive with other more specialized methods.

An increasing number of plant genetic resources (PGR) are rapidly being molecularly characterized using various marker systems (Larsen et al., 2018; Mascher et al., 2019; Milner et al., 2019). In order to effectively manage and use plant genetic resources, different methods could be employed to select a core collection (Jeong et al., 2017). Harnessing marker information to select core collections based on aspects of genetic diversity such as pairwise dissimilarity, allelic richness, or heterozygosity is feasible today (van Heerwaarden et al., 2013). Core collections use distance metrics to quantify the similarity of two accessions, based on genetic marker data or phenotypic traits (De Beukelaer et al., 2018). Different distance metrics or traits can be applied to generate core sets that are specific for a particular purpose e.g. maximizing the genetic diversity in a trait of interest. The Core Hunter software is a core set selection tool known for its flexibility to sample diverse, representative subsets from large germplasm collections with minimal redundancy (http://www.corehunter.org). Three different main versions of Core Hunter have been released. Core Hunter 3 was introduced by De Beukelaer et al. (2018) as a multi-purpose tool for selecting core subsets. For this purpose, Core Hunter 3 uses local search algorithms to provide subsets based on several distance metrics and allelic abundance. The software is capable of combining distances, entry-to-nearest-entry (E-NE) and accession-to-nearest-entry (A-NE) computations (De Beukelaer et al., 2018). Based on genetic markers, genetic differences between genotypes are calculated to evaluate the core subsets. Different methods for calculating distances are implemented. The user can either provide a genetic distance matrix which is estimated using a suitable measure such as Modified Roger's distance (Wright, 1984). On the other hand, the user can provide phenotypic traits, which are then evaluated with Gower's Distance to derive a phenotypic distance matrix (Gower, 1971).

However, for the selection of core collections, there are general clustering methods, i.e. hierarchical and partial clustering using different subtypes and algorithms to identify clusters (Kaski, 1997). Here, the focus is on partial clustering. Partial clustering comprises two clustering approaches: k-means (MacQueen, 1967) and *k*-medoids (Kaufman and Rousseeuw, 1987). *K*-medoids is known as a modified version of k-means. Both methods minimize the distance between data points within a cluster to the respective cluster center (Block et al., 2019). The main difference between methods lies in the handling of the cluster centers: While in *k*-medoids the cluster center needs to be a real object of the collection, the cluster center is an average of all cluster members in k-means and does not need to be a real object of the collection. To distinguish the two types of cluster centers, they are either called medoids (*k*-medoids) or centroids (*k*-means). Usually, *k*-medoids is considered the more robust algorithm in terms of clustering, as it is less sensitive to outliers compared to *k*-means (Park et al., 2006; Park and Jun, 2009). *K*-medoids has been used in various applications: in genetics (Broin et al., 2015), in geography (Bernábe-Loranca et al., 2014), in analyses to predict the popularity of television programs (Zhu et al., 2017), and as a decision support system in the fashion industry (Monte et al., 2013). The availability of genotypic information for different genotypes allows clustering the genotypes based on similarity or dissimilarity.

High-throughput technologies, such as next generation sequencing (NGS) or array-based technologies, offer the possibility of generating comprehensive genotype data for entire plant genomes in a short time and with high accuracy (Varshney et al., 2009). Such genotype information is also frequently used to identify marker–trait association in quantitative trait locus (QTL) mapping and genome wide association studies (GWAS) (Wang et al., 2014). The development of single nucleotide polymorphism (SNP) data has significantly increased the knowledge of genome diversity. On the other hand, advances in NGS reduced the cost of DNA sequencing, which made genotyping-by-sequencing (GBS) possible for species with high diversity and large genomes (He et al., 2014).

Several genotyping array based platforms for wheat have been published (Ganal et al., 2019). First, Cavanagh et al. (2013) developed a 9K Illumina iSelect SNP array with 9,000 SNPs. In 2014, Wang et al. (2014) reported a 90K Illumina iSelect SNP array based on the 9K array technology. The third array based platform for wheat genotyping was the Affymetrix Axiom 820K SNP array presented by Winfield et al. (2016). With this array it was possible to genotype not only hexaploid wheat but to detect and track introgressions from different sources. A subset of the markers used on this 820K array were then used to develop the Axiom 35K SNP array (Allen et al., 2017), which was specifically targeted at the elite wheat germplasm. Here we present the 15K array, a new and optimized platform containing a set of 12,908 optimized SNP markers mainly originating from the 90K chip design. This subset offers a cost-effective alternative to the 90K array.

In this paper two different methods, namely *k*-medoids and Core Hunter 3, were applied to select different sizes of core collections from a large set of wheat genetic resources and were compared to identify the most appropriate method.

## Material and Methods

### Development of the 15K Wheat Infinium Array

The 15K wheat Infinium array has been developed mainly based on genotyping data for more than 2,000 wheat genotypes consisting of European and world-wide lines, that have been generated with the 90K wheat Infinium array (Wang et al., 2014) at TraitGenetics. The selection steps that were applied to create the 15K array are as follows:

1. Based on the raw genotyping data, all markers were surveyed for marker quality during the cluster file development using the Illumina GenomeStudio software (Illumina, San Diego, USA). Markers with clearly differentiated clusters were identified independently whether the markers were genome-specific (Ganal et al., 2012).
2. Genetic mapping data (Wang et al., 2014) were used together with additional mapping data generated from the ITMI DH population (Sorrells et al., 2011) for selecting markers that are evenly distributed throughout the genetic map of the three (A, B, D) wheat genomes.
3. Using the marker order determined by the genetic mapping, additional markers were integrated in case they were in perfect linkage disequilibrium with at least one other mapped marker.
4. Haplotype blocks were defined as containing markers in perfect linkage disequilibrium over all investigated wheat lines. From each larger haplotype block especially in the centromeric regions, one or two markers were selected based on the marker quality defined by Wang et al. (2014).
5. The markers from the 90K array were supplemented by 383 additional markers from an unpublished 12K wheat Infinium array previously developed by TraitGenetics for haplotype blocks that were not identified using the 90K markers.
6. Finally, a set of 27 public markers derived from candidate genes for major wheat phenology traits has been added.

In total, 15,000 markers were submitted for array design to Illumina of which 12,908 markers remained after array manufacturing and an additional genotyping round of 384 wheat lines to identify low quality markers. These were used for the development of a cluster file for allele calling. These functional markers are listed in **Supplementary Table S1** which also includes information about the origin (90K or 12K or candidate gene) and the respective context sequence.

### Plant Material

In this study, a collection of 890 winter wheat genotypes was used for the development of a small genetically diverse core collection. The 890 genotypes were collected from five different collections, which had been used in different studies at the Julius Kuehn Institute, Federal Research Centre for Cultivated Plants, Institute for Resistance Research and Stress Tolerance (JKI-RS). Ninety two were evaluated under drought stress and well-watered conditions in the presence and absence of mycorrhizae to identify QTLs involved in response to mycorrhizae under drought stress condition (collection 1) (Lehnert et al., 2018). Babben et al. (2018) and Soleimani et al. (in preparation) evaluated 284 genotypes to identify genome regions associated with frost tolerance (collection 2). A set of 40 genotypes was tested for resistance against soil borne viruses (collection 3). These three collections were genotyped by using the 90K Illumina iSelect array (Wang et al., 2014), with the exception of five genotypes from collection 3, which were genotyped using the 15K Infinium array. Furthermore, 220 genotypes were evaluated under two different nitrogen concentrations [collection 4, (Voss-Fels et al., 2019)], and 254 genotypes were inoculated with wheat dwarf virus to select genotypes tolerant against this virus (collection 5), respectively. These genotypes were genotyped by using the 15K Infinium array.

As two different platforms (15K and 90K) were used for genotyping the wheat genotypes, only common markers (markers which were detected by the 15K and 90K array approach) were used for further analyses. A principal coordinate analysis (PCoA) was conducted with the package ‘ape' (Paradis and Schliep, 2018) in the R statistical environment based on the Modified Roger's distance (MRD) matrix to visualize the genetic diversity in the five collections.

### Placement of SNP Array Marker Sequences Onto the Pseudomolecule Reference Sequence

The published reference genome of the bread wheat cultivar Chinese Spring (the IWGSC RefSeq) and the genome annotation were downloaded (Appels et al., 2018). SNP array marker sequences were split at the polymorphic site with a custom awk script and turned into paired-end style sequencing reads, effectively reverse complementing one of the reads. These artificial paired-end reads were then mapped to the bread wheat pseudomolecule reference sequence with BWA mem (version 0.7.13) with -M parameter for highlighting of secondary alignments (Li and Durbin, 2009; Li, 2013). Alignments were converted to BAM format with SAMtools (version 1.6) (Li et al., 2009). Unmapped reads and secondary alignments were discarded and remaining high quality alignments (MAPQ ≥ 20) were transformed to BED format with BEDtools (version 2.8) keeping the CIGAR string (Quinlan and Hall, 2010; Quinlan, 2014). Filtered alignments were then checked for consistency with a custom Java program. Briefly, reads without a mapped mate, pairs of reads that do not map exactly one nucleotide apart, and mapped reads where the SNP position was an unknown nucleotide (‘N') were removed. Afterwards, all mapped markers were evaluated on the 890 genotypes. Markers with equal or more than 30% of missing data as well as monomorphic markers were removed from further analysis. Duplicate markers and markers mapping to the same physical position were removed as well and only the initial marker was kept. The filtered marker data were used for SNP imputation by applying the software package Beagle version 4.1 (Browning and Browning, 2007; Browning and Browning, 2009). Imputed marker data were filtered for minor allele frequency (MAF) ≥ 5%, and heterozygosity ≤ 12.5%, resulting in a set of 7,672 SNP markers used for subsequent analyses.

### *K*-Medoids Clustering

Based on the Modified Roger's distance (MRD) matrix, 890 genotypes were clustered into 178 and 320 groups by using the *k*-medoids clustering method (Kaufman and Rousseeuw, 1987). *K*-medoids clustering was conducted by using the cluster package (version 2.1.0) and PAM method in the R statistical environment (Maechler et al., 2012; RDevelopment CORE TEAM, 2015).

### Core Hunter 3

Two different genetic distances, 1) MRD (Roger, 1972; Wright, 1984), 2) and Cavalli-Sforza and Edwards (CSE) distance (Cavalli-Sforza and Edwards, 1967) were applied to calculate different core sets. In total, 14,000 different core sets were determined (two sizes times seven different settings times 1000 iterations in Core Hunter 3). Different approaches for calculating core sets in Core Hunter 3 were used, *i.e.*:

- Average Entry-to-Nearest-Entry distance (E-NE) (Odong et al., 2013): This is the mean distance between all selected accessions and their closest other selected accession. Maximizing this measure yields high diversity in the core collection expressed through maximum dissimilarity of selected core accessions (De Beukelaer et al., 2018). Both genetic distances (MRD and CSE) were applied for calculating these core sets.
- Average Accession-to-Nearest-Entry distance (A-NE) (Odong et al., 2013): The A-NE considers the mean distance between each accession in the whole collection and the closest selected accession. Minimizing this measure yields core collections that maximally represent all individual accessions from the full collection (De Beukelaer et al., 2018). Both genetic distances (MRD and CSE) were applied for calculating these core sets.
- Shannon's diversity index (Shannon, 1948): Shannon's diversity index is an appropriate measure when forming core subsets that attempt to retain as many rare alleles as possible, regardless of their co-location within loci (Thachuk et al., 2009). The Shannon diversity index achieves its highest value when each allele exists only once in the whole data set being measured.
- Expected heterozygosity (Berg and Hamrick, 1997): The expected proportion of heterozygous loci on the other hand, specifically considers diversity within each locus. Intuitively, since each locus contributes equally to the overall value of this measure, core subsets selected using this measure are less likely to be homozygous for a number of different loci than core subsets selected with Shannon's Diversity index (Thachuk et al., 2009).
- Allele coverage: The percentage of marker alleles observed in the full collection that are retained in the core. This is a simple measurement, which indicates the percentage of retained alleles in the core set relative to the whole population. This method is particularly useful for selecting core sets to preserve alleles in gene and seed banks (Thachuk et al., 2009).

## Results

The overlap between the 15K and 90K arrays resulted in 8221 SNP markers that could be mapped to unique positions in the reference wheat genome sequence. Of these markers, the majority (45%) mapped to sub-genome B, followed by sub-genome A with 39%, while the lowest proportion (15%) was located on sub-genome D. Less than 1% of markers were mapped to sequences located to chromosome ‘unknown', an artificial chromosome consisting of sequences that could not be assigned to any chromosome yet. Among the chromosomes, the highest and lowest number of mapped markers was identified on chromosomes 5B and 4D with 595 and 62 markers respectively. The number of mapped markers per chromosome is listed in **Table 1**. To understand the effects on observed versus expected heterozygosity based on the array system, a set of 48 wheat accessions was analyzed by genotyping them with the 15K and 90K array. During this comparison no significant differences between array systems was detected (**Figure S1**).

**Table 1:** Distribution of uniquely mapped markers on the reference genome sequence from the 15K SNP array.

Chromosome	Wheat genome			Total
	A	B	D	
1	451	580	270	1,301
2	480	710	289	1,479
3	415	556	142	1,113
4	287	258	62	607
5	508	595	186	1,289
6	485	530	169	1,184
7	546	485	144	1,175
Total	3,172	3,714	1,262	8,148
Unknown	73			8,221

**Figure 1:**
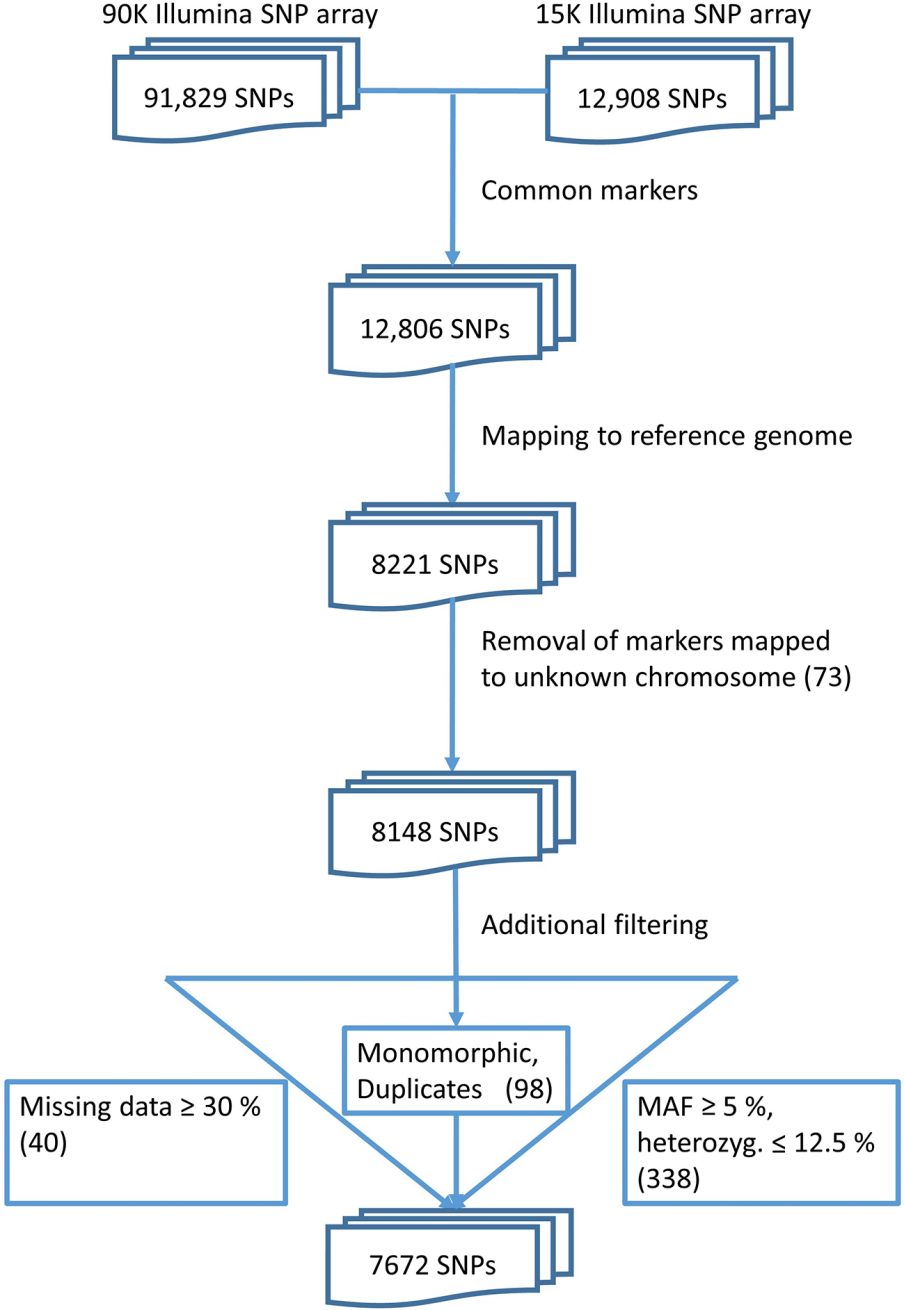
Data pre-processing finally yields a set of 7,672 markers.

The quality check of the markers resulted in a set of 7,672 polymorphic, informative markers (**Figure 1**). These markers were placed at unique positions on the reference genome sequence of bread wheat (*cv.* Chinese Spring). This final set of markers was used for further analyses.

Furthermore, a principal coordinate analysis (PCoA) was performed (**Figure S2**). The first and the second principle coordinates (PCs) explained 9.5 and 4.2% of the total variance and were used to graphically display the results. The analysis showed that most genotypes from the different collections were not clearly separated from each other. Although clusters of genotypes from collections can be observed, outliers from each collection can also be found near or within clusters of other collections. Most genotypes belong to the collections 2 and 5.

### Comparing Different Core Sets

In total, 178 and 320 genotypes were selected by *k*-medoids clustering and Core Hunter 3, respectively. Core Hunter 3 uses random seeds and a non-deterministic algorithm to arrive at a solution after a time (or alternatively step) threshold has been reached. Similarly, the *k*-medoids algorithm as implemented in the PAM function inside the R library ‘cluster' also works non-deterministic. However, in the so-called build phase the program chooses a good initial set of medoids. In our tests given our population and MRD matrix, it always produced the same core collection. Therefore, we randomly sampled initial medoids and gave these to the PAM function as input parameters allowing to compare the stability of the obtained results with those from Core Hunter 3.

Our goal was to assess the results obtained through a large number of iterations (n = 1000) to get information on 1) the stability of the methods, 2) the influence of the size of the core collection size, and 3) which method performs best for the two main objectives to form core collections: CC-I and CC-X.

**Figure 2:**
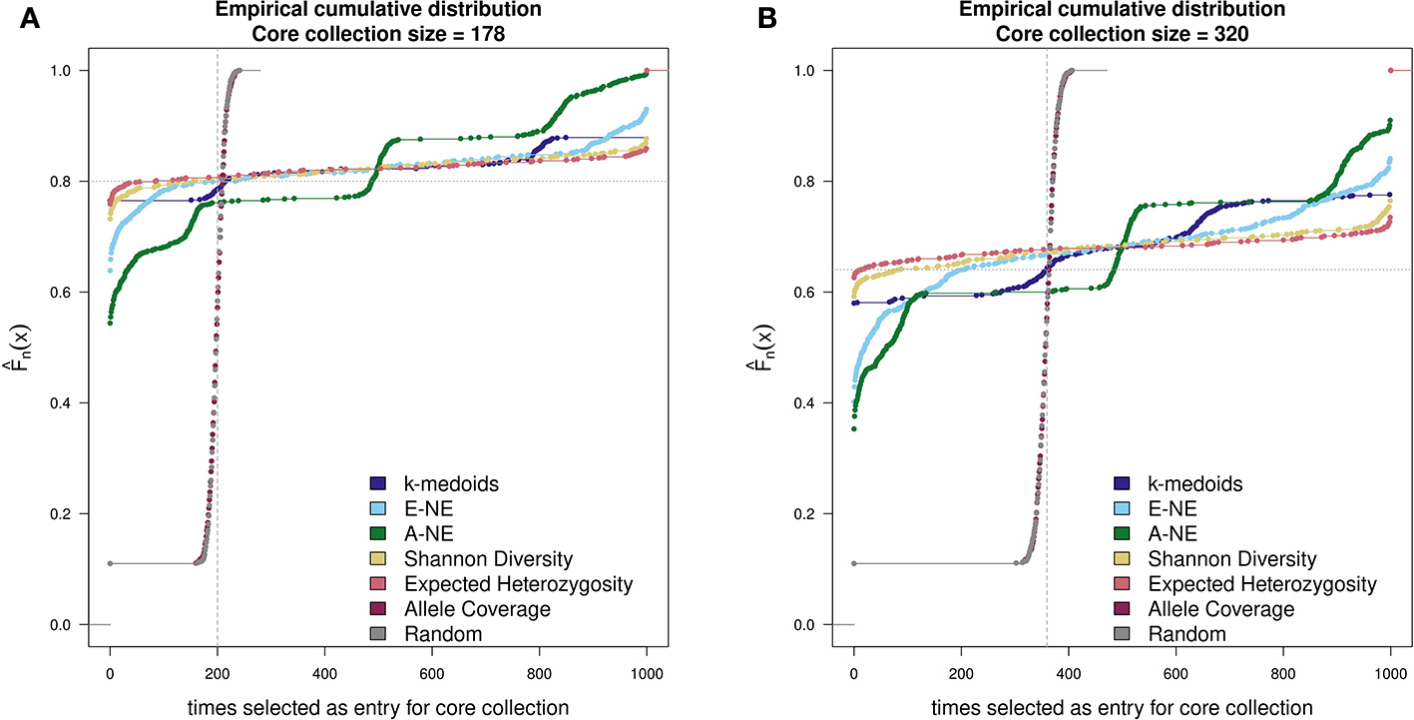
Comparison of stability test with 1,000 runs between *k*-medoids derived core set, seven core sets derived by Core Hunter 3 and randomly selected core sets. **(A)** depicts the stability results for the core set containing 178 genotypes, while **(B)** depicts the stability results for the core set containing 320 genotypes. Methods with a low gradient are considered to be stable; large gradients, on the other hand, show a high degree of variability. Two gray helper lines have been added for easier visual interpretation of results. The dotted horizontal line indicates stable results, while the dashed vertical lines shows instability.

For testing the stability of the different methods implemented in Core Hunter 3 and *k*-medoids we performed an empirical cumulative distribution analysis with the function ‘ecdf' in the R statistical environment. We evaluated the resulting core sets by looking at the composition of entries in 1,000 runs per method and two different core set sizes (178 and 320). For the goal of observing the gain from using any core selection program, we also constructed 1000 random sets per core set size using the R function ‘sample'. The stability results for all tested methods demonstrated similar behavior for both core set sizes of 178 and 320 genotypes (**Figure 2**). Taking into account the definition of stability (Higham, 2002; Atkinson and Han, 2005; Soleimani and Weiner, 2018), a method returns stable results if all genotypes are either never or always selected. In contrast a method returns unstable results if all genotypes are uniformly selected. However, stability is not a binary feature, it is much more continuous. The stability test was characterized by the frequency of a genotype selected by a method as an entry into a core collection. The ecdf of a stable method should be close to the grey horizontal dotted line, while the ecdf of an unstable method should be close to the grey vertical dashed line. Based on the observed results, Shannon's diversity and expected heterozygosity in all 1,000 runs showed a high number of entries in the core sets that were common between runs and can therefore be considered stable methods (**Figure 2**). On the basis of the stability analysis we obtain a ranking of the applied methods according to increasing stability: A-NE, E-NE, *k*-medoids, Shannon's diversity and expected heterozygosity. Both the random and allele coverage sets, on the other hand, showed a very unstable behavior.

**Figure 3:**
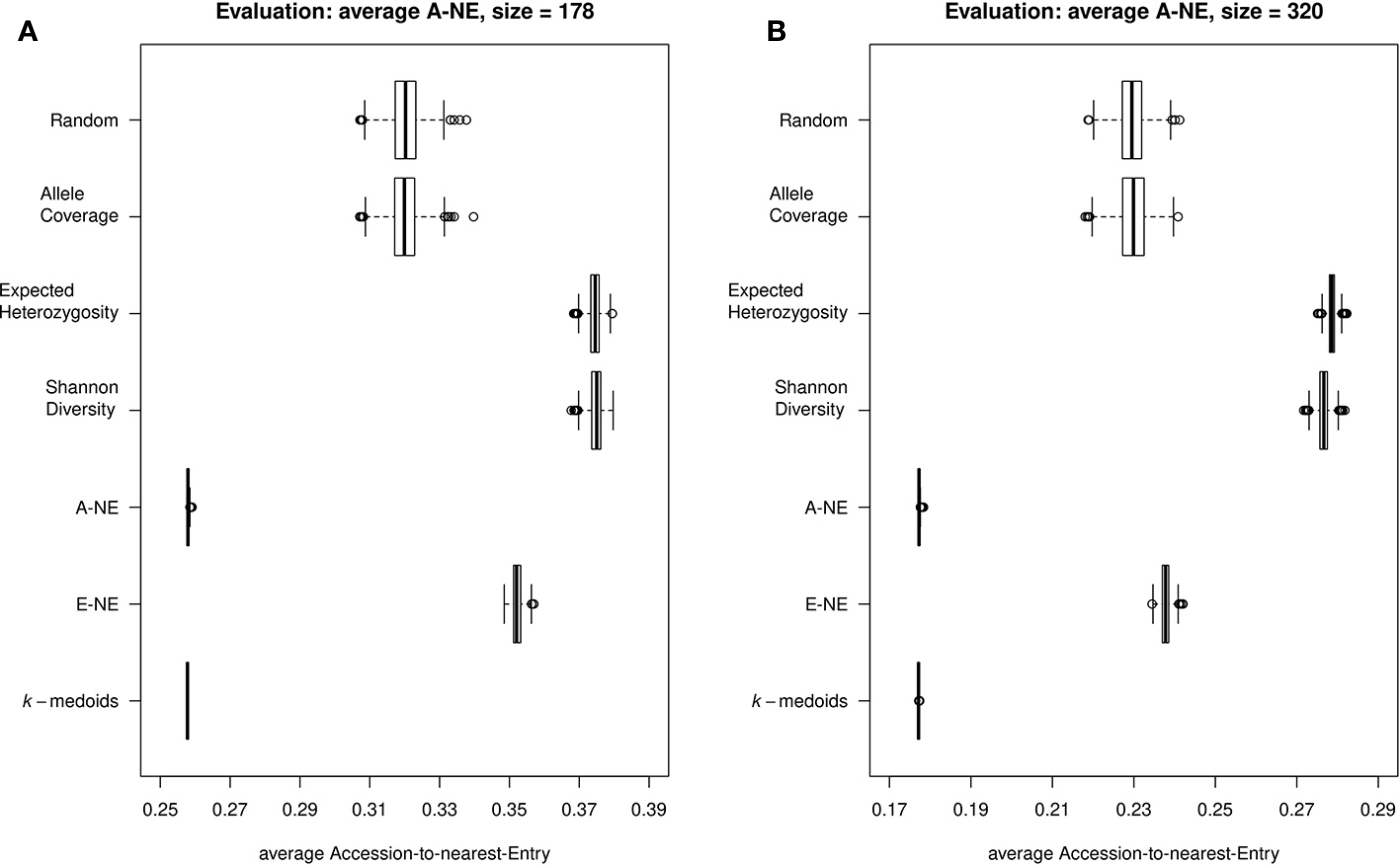
Quality of core collections. Displayed are the average distances between each of the 890 accessions to the nearest entry of the respective core set (A-NE) for core collections of different sizes. **(A)** shows core sets of size 178, while **(B)** shows core sets of size 320. A low average distance is favorable to obtain a good representation of the original collection.

To evaluate the quality of selected core sets, we calculated two average distances as proposed by Odong et al. (2013). The average A-NE result varied between 0.25 to 0.39 and 0.17 to 0.29 for a core set size of 178 and 320 genotypes, respectively (**Figures 3A, B**). The lowest average A-NE was observed for *k*-medoids, and also the average Accession-to-Nearest-Entry method (A-NE) showed low values for average A-NE. Both Shannon's diversity (SD) and expected heterozygosity (EH) showed high values for average A-NE and therefore performed worse compared to the other methods. Based on results obtained for average A-NE, the methods *k*-medoids and A-NE were best suited to represent the original population due to the smallest value for average A-NE (**Figures 3A, B**) for both sizes of core sets.

**Figure 4:**
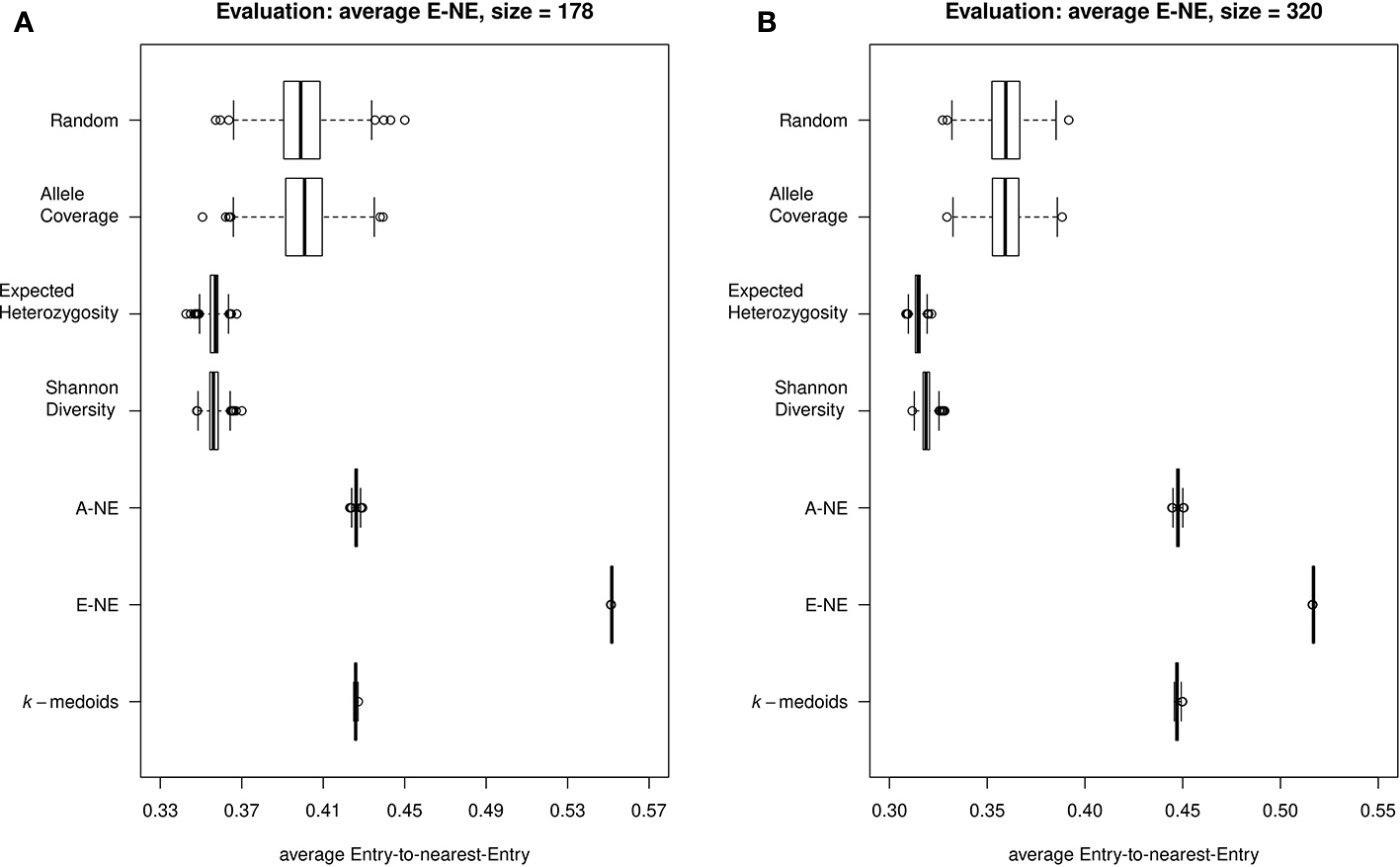
Quality of the core sets. Displayed are the average distances between each of the entries to the nearest entry of the respective core set (E-NE) for core sets of different sizes. **(A)** shows core sets of size 178, while **(B)** shows core sets of size 320. A high average distance is favorable to obtain a good representation of the extreme genotypes of the original collection.

Furthermore, our results for average E-NE calculation for both sizes of core sets showed that the method based on Entry-to-Nearest-Entry distance (E-NE) performed better to represent extreme genotypes compared to other core sets, as the obtained average E-NE showed the highest value among all analyzed core sets (**Figures 4A, B**). The methods based on Shannon's diversity (SD) and expected heterozygosity (EH) showed the lowest values for the average E-NE. Therefore, based on observed results, two core set methods (SD and EH) indicate an insufficient representation of the extreme genotypes from the original population in both sizes of core sets.

**Figure 5:**
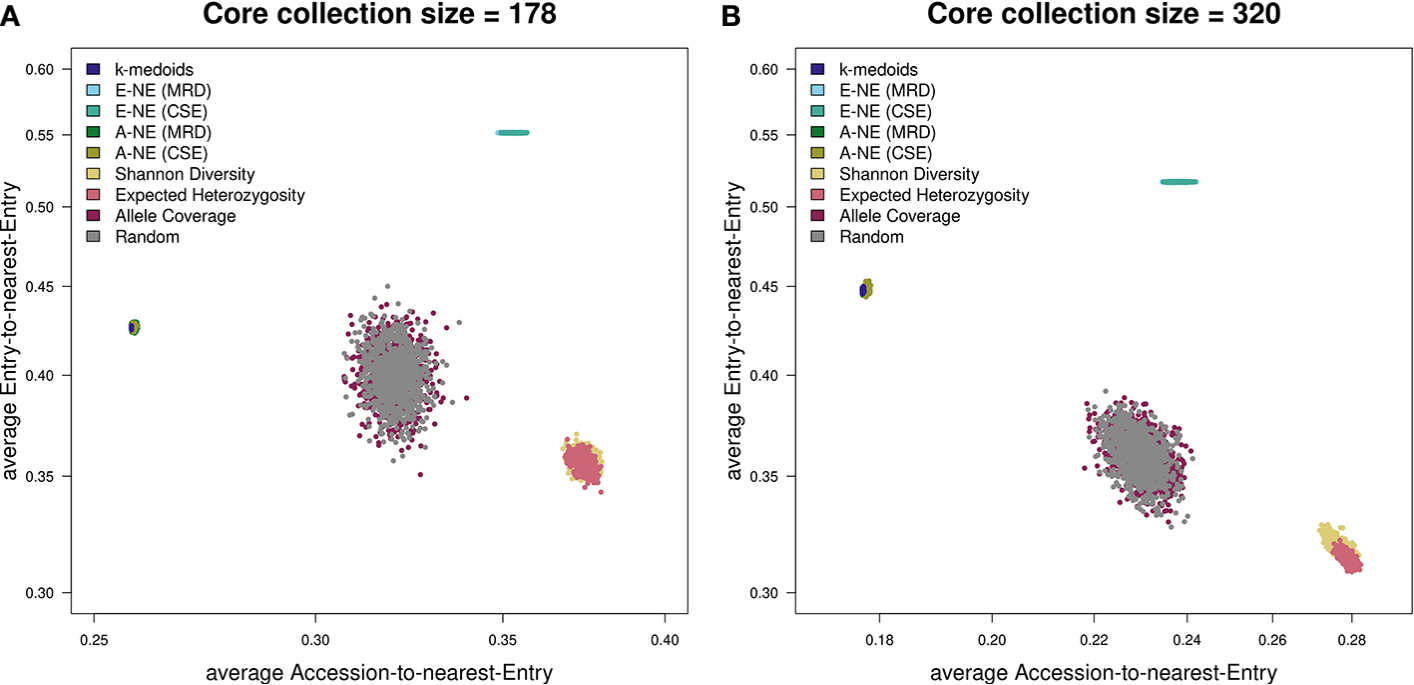
Scatterplots showing both average A-NE and average E-NE for the observed core collections for different sizes. **(A)** shows core collections of size 178, while **(B)** shows core collections of size 320. As already indicated by the stability test (**Figure 2**), the core collections from the type allele coverage show a large variance in their distribution. The Shannon diversity and expected heterozygosity methods seem to produce core collections of similar quality. The same seems to be true for *k*-medoids and A-NE methods. A theoretically optimal core collection would be located in the upper left corner of the plot.

The two genetic distance metrics, MRD and CSE, that were used for the two core selection methods A-NE and E-NE produced very similar results throughout the different evaluations (**Figure 5**) and for the sake of simplicity only the results obtained by using MRD are shown in **Figures 2**–**4**.

## Discussion

The development and use of molecular markers has expanded our knowledge to better understand cereal genetics. High-throughput SNP array genotyping allows genotyping thousands of markers in parallel. This technique has been applied in recent years for small grain cereals such as barley, wheat, rye, and oats (Ganal et al., 2019). The 90K Illumina Infinium array is currently the most widely used genotyping array in wheat. However, this genotyping array is quite expensive on a price per sample base and creates a large set of redundant data (Ganal et al., 2019). Subsequently, the Affymetrix Axiom 820K SNP array was developed to genotype wheat and to detect and track introgressions. Later, this technology was used for the development of the Axiom 35K SNP array. In this study, we also used the new 15K Illumina Infinium array with 12,908 functional markers that contains mainly high quality and informative markers. The overlap between the two array platforms (15K and 90K) is 12,806 markers. The 15K genotyping array with a lower number of markers is a cost-effective option for genotyping experiments that still provides high resolution data.

Breeders seek to improve yield performance by exploiting favorable traits associated with tolerance against biotic and abiotic stress (Pandey et al., 2017). Germplasm collections from major crops have increased in size and number worldwide (Brown et al., 1997). Genebanks play an important role in securing genetic diversity for future use. They are distributed around the world and preserve the genetic diversity in crop species (Shands, 1990; Fowler and Hodgkin, 2004).

The increase in the size of germplasm collections leads to problems and complications in the characterization, evaluation, utilization and maintenance of germplasm. The first approach to reduce the size of large collections and to select core sets of these collections was defined by Frankel (1984). Core collections became important due to the demand for more efficiency in the characterization and utilization of collections stored in genebanks (Odong et al., 2013). Different methods are available to create core collections for varying purposes with respect to phenotypic and genotypic data. These methods could be used to select genetically diverse genotypes for carrying out different scientific research before a large number of genotypes are phenotyped, thus excluding genotypes that would show the same behavior. Therefore, by eliminating the need for an additional phenotyping step, these approaches could accelerate research experiments and breeding programs. Molecular markers are widely used to unlock the genetic diversity of germplasm collections. Odong et al. (2013) pointed out the role of genetic differentiation in marker data, which has a significant impact on core selection methods.

Different algorithms are known for the generation of core sets, and comparisons between different algorithms have been made in previous studies. For example, Thachuk et al. (2009) compared three different algorithms (D-method, MSTRAT and PowerCore) with Core Hunter to select core sets in a maize population. The comparisons confirmed that Core Hunter performed better than other methods in creating core sets with higher genetic diversity. Also, Core Hunter was able to select significantly smaller core subgroups that retained all unique alleles from an original collection than the other algorithms. In our study, we used the same genetic distance and genetic diversity indices as Thachuk et al. (2009) to compare *k*-medoids and Core Hunter 3 for core collection selection.

In the present study, we conducted a stability test for six methods comprising allele coverage (AC), expected heterozygosity (EH), Shannon's diversity (SD), A-NE, E-NE and *k*-medoids to analyze their reproducibility. Based on the definition of Higham (2002) and Atkinson and Han (2005), SD and EH, were more stable than other methods. A-NE and E-NE methods provided by Core Hunter 3 as well as *k*-medoids can be classified as stable methods for the selection of core collections. AC showed a highly unstable performance when selecting core sets and should be avoided when core sets should be reproducible (as it also highly resembled the random selected sets).

In the present study, two genetic metrics were applied to assess the quality of different core set selection methods (Odong et al., 2013). For the evaluation of CC-I core sets, the calculation of the average A-NE is a suitable method. For such an objective the average A-NE value should be as small as possible. An average A-NE value equal to zero indicates a minimal distance between genotypes and thus the maximum representation of the genotype in the core collection. Based on this definition, the *k*-medoids and A-NE derived core sets did the best job to achieve maximum genetic diversity of genotypes with the lowest average value of A-NE observed. On the other hand, a good criterion for the evaluation of CC-X core sets is to maximize the average E-NE. The E-NE method describes how genetically diverse the entries into the core set are to each other. Therefore, the best possible core set for CC-X strategy has the highest average E-NE. In our tests, the average Entry-to-Nearest-Entry (E-NE) core collections compared to other core set methods performed best in this category. However, it is not surprising that A-NE derived core collections yield good results for CC-I and E-NE derived core collections yield good results for CC-X.

For a final assessment of core selection methods, we evaluated and combined the results of the stability test and the quality of core selection on the basis of average A-NE/E-NE. Based on the stability tests, the most stable core selection methods are Shannon's diversity (SD) and expected heterozygosity (EH). While these two core selection methods showed less good results for the average A-NE and the average E-NE for different purposes (CC-I and CC-X) of core collections, they should therefore not be considered superior to the other core selection methods. Although *k*-medoids is a general clustering method and is not specifically designed for creating core collections, it proved to be one of the better methods for creating CC-I core sets due to its small average A-NE value. Based on our results from the evaluation with average E-NE, *k*-medoids also proved to be an adequate method for the generation of CC-X core sets. Interestingly, the A-NE based core selection methods showed very similar profiles to the *k*-medoids method in both average A-NE and average E-NE evaluation, but were somewhat more unstable in the stability test (**Figures 3**–**5**).

## Conclusion

In the present study, we used the wheat 90K Infinium array together with an optimized 15K Infinium array with 12,908 informative markers. Compared to the 90K array, the 15K array is a cost-effective platform for research and plant breeding programs that generates high quality data. We selected core collections of 178 and 320 genotypes from a collection of 890 wheat genotypes using *k*-medoids and Core Hunter 3. Two genetic distances and three indices of genetic diversity were used to establish core collections and the results were compared to determine the best approach for a large population of diverse genotypes. Our results support the conclusion that choosing either MRD or CSE as genetic distance has little to no observable effect on the selection of core collections using A-NE and E-NE in Core Hunter 3. In addition, *k*-medoids and Accession-to-Nearest-Entry (A-NE) are appropriate methods to select a uniform representation of the original population (CC-I). However, if the purpose of generating a core collection is to construct a core set based on the extremes of the relevant traits (CC-X), the method Entry-to-Nearest-Entry (E-NE) showed the best results. Furthermore, both *k*-medoids and A-NE methods seem to be a good compromise when trying to combine the goals of CC-I and CC-X (**Figure 5**). Finally, A-NE, E-NE and *k*-medoids yield stable results if started multiple times independently.

## Data Availability Statement

The datasets generated for this study can be found in Zenodo, DOI: 10.5281/zenodo.3905912.

## Author Contributions

FO and DP conceived and designed the experiments, collected all genotypic data from five different collections for 890 genotypes of wheat. HL, JK, SN, SB, and BS performed the statistical analyses on the data. JP and MG provided the 15K array design and data. SB and BS wrote the initial draft. SB, BS, DP, HL, JK, and SN interpreted the data. All authors contributed to the article and approved the submitted version.

## Funding

This research was financially supported by a grant (project 031B0186D) from the German Federal Ministry of Education and Research, Bundesministerium für Bildung und Forschung (BMBF).

## Conflict of Interest

The authors JP and MG have competing commercial interests as members of TraitGenetics GmbH which is a company that offers marker development and analysis (including this array) for commercial purposes. This does not alter the authors' adherence to sharing all data and materials. There are no further products in development or marketed products or patents to declare.

The remaining authors declare that the research was conducted in the absence of any commercial or financial relationships that could be construed as a potential conflict of interest.

## Supplementary Material

Figure S1 Comparison of the effect of the choice of the array system on the observed and expected heterozygosity. A set of 48 wheat accessions was genotyped on both 15K and 90K Illumina Infinium arrays and both observed and expected heterozygosity were calculated. Panel **(A)** shows the observed heterozygosity (Ho) on 15K and 90K arrays, panel **(B)** shows the expected heterozygosity (He) on 15K and 90K arrays. The relative frequency of observed heterozygosity (Ho) is shown in panels **(C)** (90K array) and **(D)** (15K array), while the relative frequency of expected heterozygosity (He) is shown in panels **(E)** (90K array) and **(F)** (15K array).

Figure S2 Principal coordinate analysis (PCoA) indicating genetic diversity over five different collections for a total population of 890 wheat genotypes.
