# Supplementary figures and images for: Comparison Between Core Set Selection Methods Using Different Illumina Marker Platforms: A Case Study of Assessment of Diversity in Wheat
Source: Front Plant Sci. 2020 Jul 9;11:1040. doi: 10.3389/fpls.2020.01040 (PMC7381318; doi:10.3389/fpls.2020.01040)

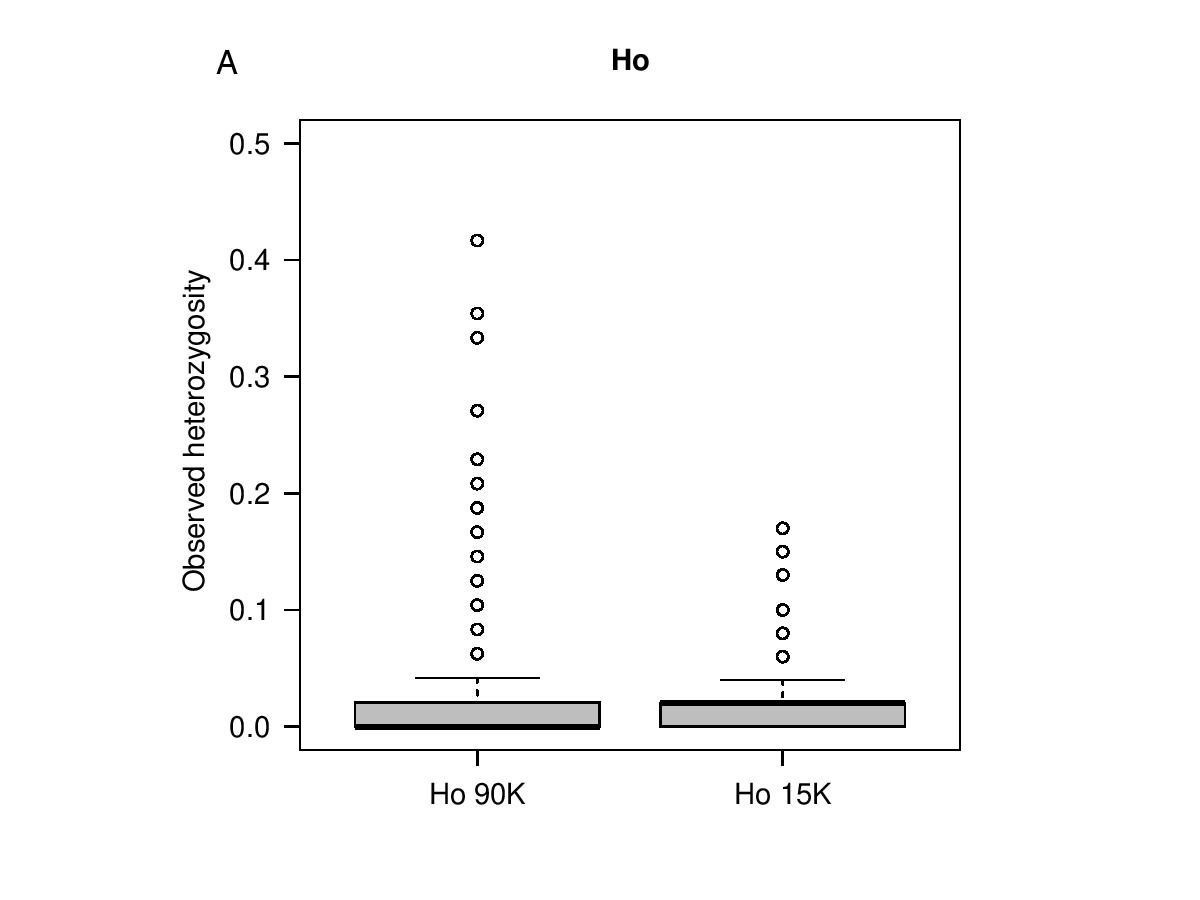

Supplement: Figure S1 — Comparison of the effect of the choice of the array system on the observed and expected heterozygosity. A set of 48 wheat accessions was genotyped on both 15K and 90K Illumina Infinium arrays and both observed and expected heterozygosity were calculated. Panel (A) shows the observed heterozygosity (Ho) on 15K and 90K arrays, panel (B) shows the expected heterozygosity (He) on 15K and 90K arrays. The relative frequency of observed heterozygosity (Ho) is shown in panels (C) (90K array) and (D) (15K array), while the relative frequency of expected heterozygosity (He) is shown in panels (E) (90K array) and (F) (15K array). [file Image_1.jpg]

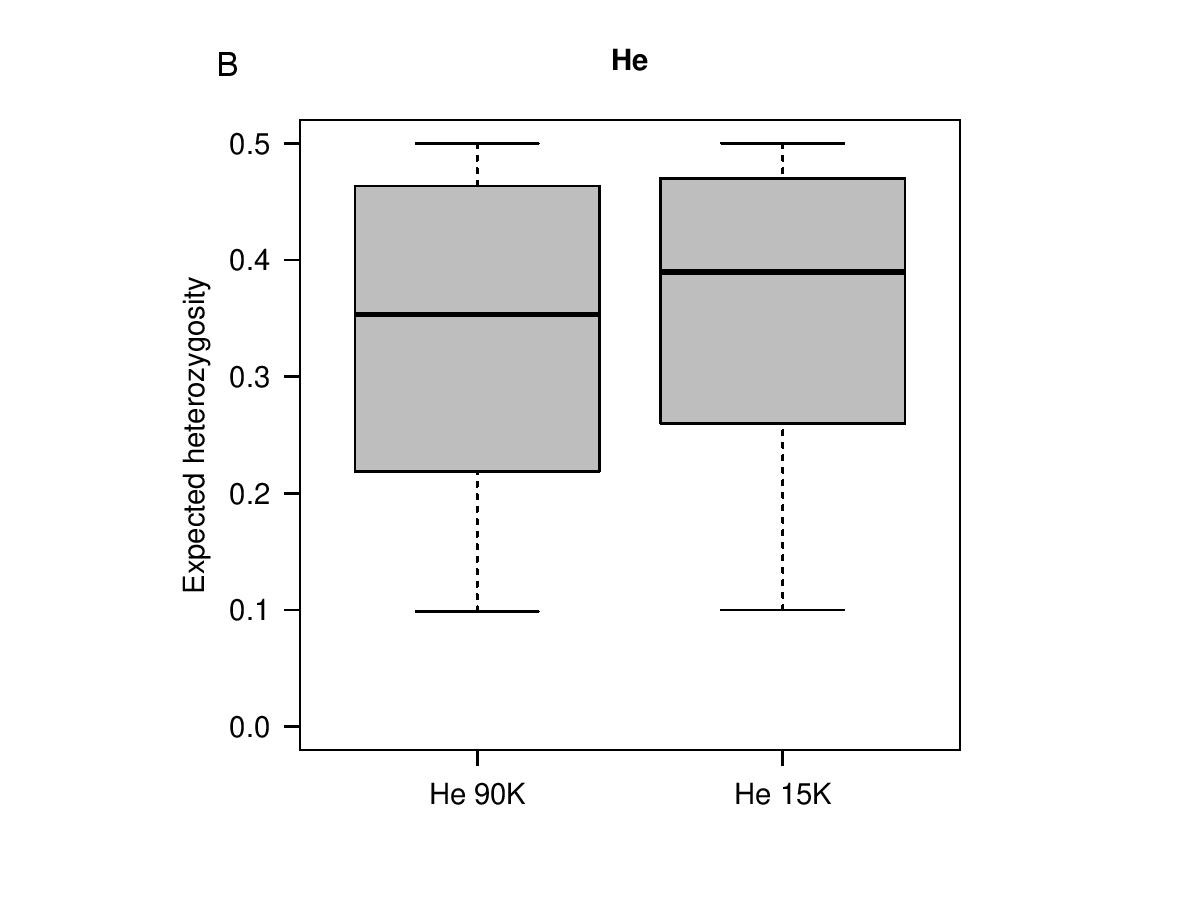

Supplement: Figure S2 — Principal coordinate analysis (PCoA) indicating genetic diversity over five different collections for a total population of 890 wheat genotypes. [file Image_2.jpg]

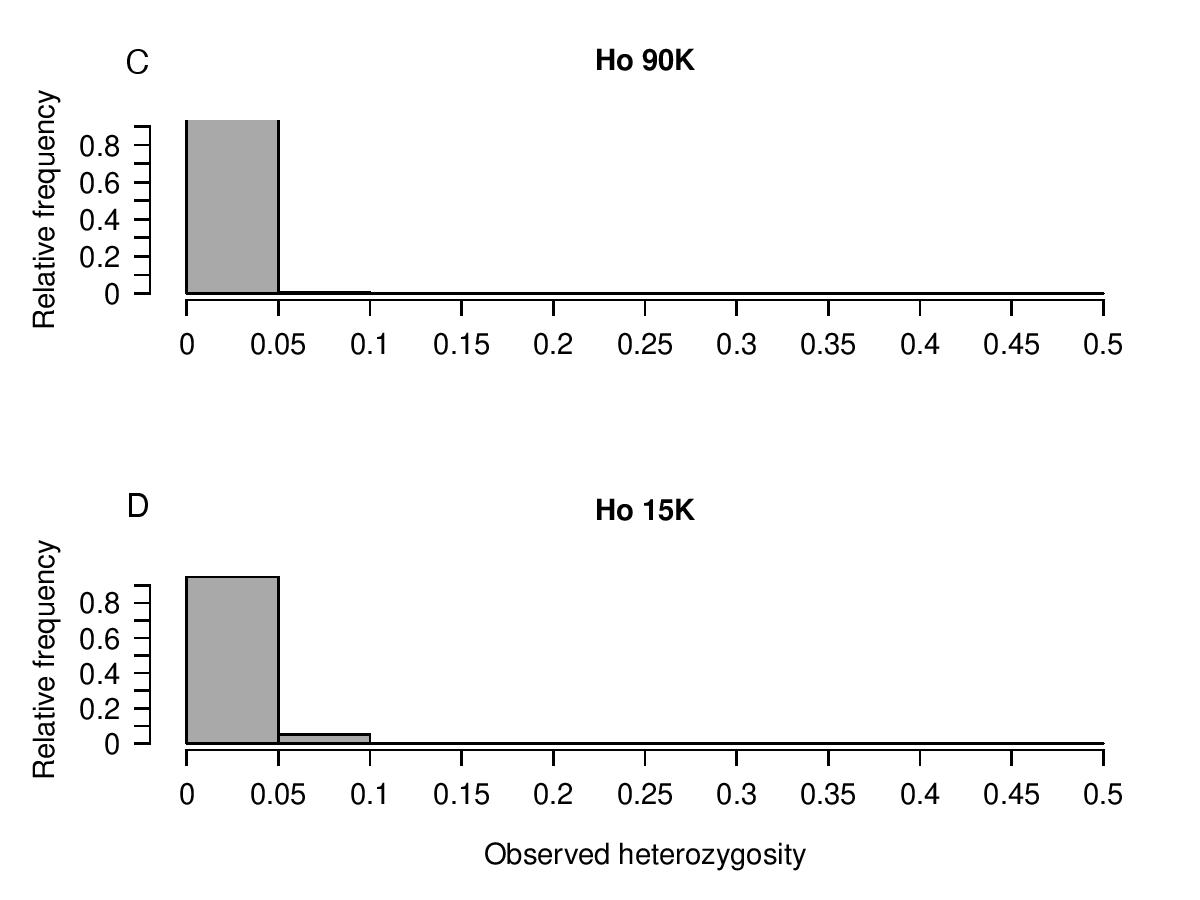

Supplement: Supplementary file 3 [file Image_3.jpg]

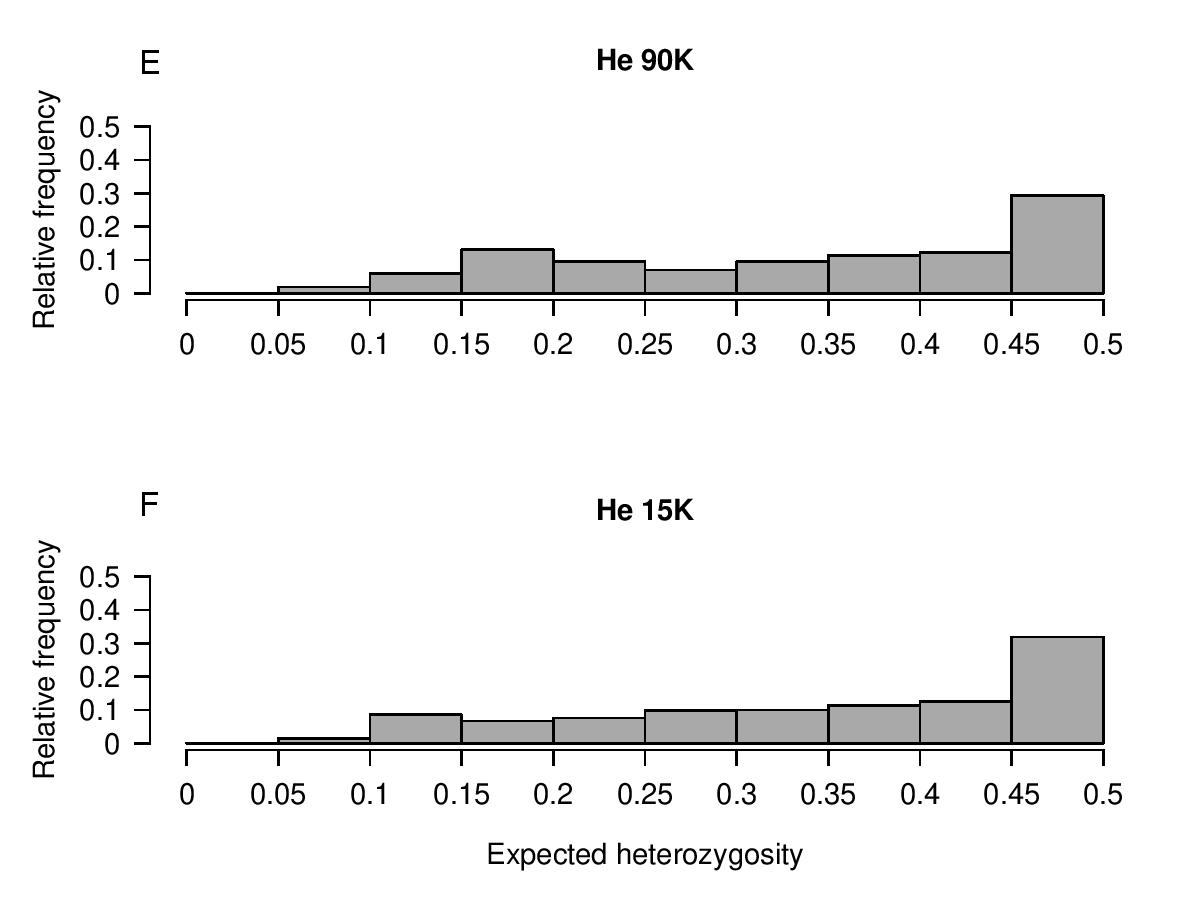

Supplement: Supplementary file 4 [file Image_4.jpg]

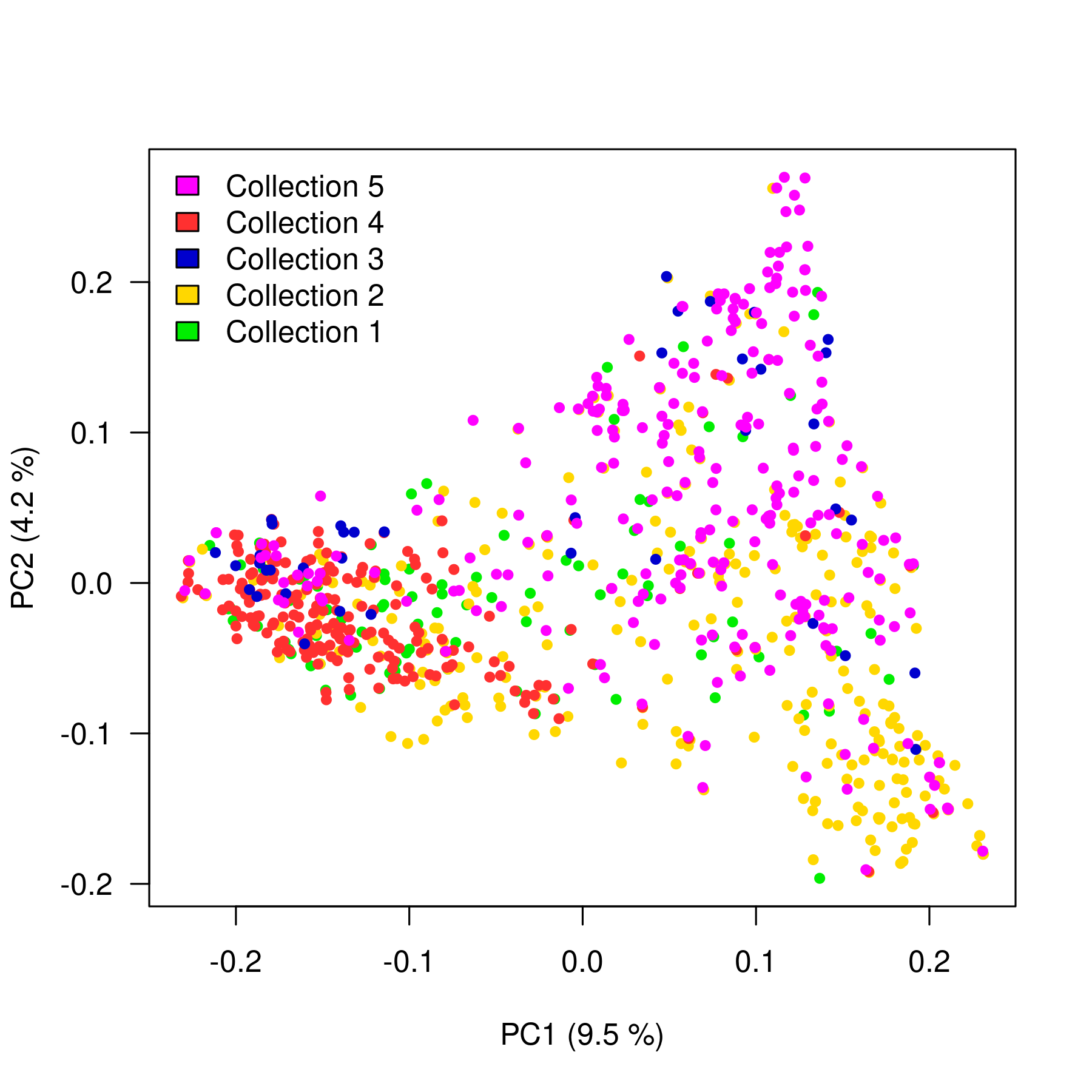

Supplement: Supplementary file 5 [file Image_5.png]
